# Optimal fishing effort for commercial catfish (*Clarias gariepinus*) fishery in the southeast arm of Lake Malawi: A Gordon-Schaefer model approach

**DOI:** 10.1371/journal.pone.0343577

**Published:** 2026-08-20

**Authors:** Francisco Chamera, Mphatso Kamndaya, Patrick Phepa, Solomon Kadaleka, Peter Mpasho Mwamtobe, Alpha Omega Soko

**Affiliations:** 1 Basic Sciences Department, Lilongwe University of Agriculture and Natural Resources, Lilongwe, Malawi; 2 Department of Mathematical Sciences, Malawi University of Business and Applied Sciences, Blantyre, Malawi; 3 Department of Applied Studies, Malawi University of Science and Technology, Blantyre, Malawi; Central Marine Fisheries Research Institute, INDIA

## Abstract

This study determines the optimal fishing effort for the commercial catfish (Clarias gariepinus) fishery in the southeast arm of Lake Malawi, with the aim of maximising economic benefits while maintaining a biologically sustainable fish stock. The study utilised secondary catch and effort data covering the period 2000–2023, together with secondary fish price and fishing cost data derived from the same previously published study. The Gordon–Schaefer (GS) model was used to estimate the static exploitation reference points of maximum sustainable yield (MSY), maximum economic yield (MEY), and open-access yield (OAY). Bifurcation analysis of the GS model showed that, for the continued persistence of the resource, fishing effort should remain below the bifurcation threshold of *E* = 6830 trips per year. The study further discusses the optimum sustainable yield (OSY), a dynamic exploitation level. Analysis of the exploitation reference points showed that the optimal fishing effort is approximately 445 trips per year, corresponding to the MEY reference point, which coincides with the OSY effort when the discount rate is zero. Since the observed fishing effort substantially exceeds 445 trips per year, the study recommends implementing management strategies aimed at reducing fishing effort to sustainable levels in order to ensure long-term biological and economic sustainability of the fishery.

## Introduction

The fisheries sector in Malawi plays a significant role in supporting local livelihoods by contributing to the social and economic well-being of the population. It serves as an important source of nutrition, income, and employment. Approximately 60% of the dietary animal protein intake and 40% of the total protein supply in Malawi are derived from fish [1]. The sector directly employs about 60,000 fishers and indirectly supports approximately 500,000 people involved in fish processing, fish marketing, net making, boat building, and engine repairs [2]. In addition, it generates approximately USD 24 million annually, contributes around 4% to Gross Domestic Product, and supports import substitution since most fish consumed in the country is locally produced [1]. These contributions highlight the need for effective management of fishery resources to ensure long-term sustainability.

Lake Malawi is the third-largest lake in Africa and the ninth-largest in the world. It hosts a highly diverse fish fauna, with more than 1,000 species, many of which are endemic [3]. The lake therefore represents a critical ecological system as well as a major resource for fisheries production and scientific research.

In Malawi, the fisheries sector is broadly classified into capture fisheries, aquaculture, and the aquarium trade. Capture fisheries dominate, contributing approximately 90% of total fish production. This subsector is further divided into artisanal (small-scale) and industrial (commercial) fisheries. Artisanal fishers typically operate small vessels such as planked boats and dugout canoes, while industrial fishers use stern trawlers and pair trawlers. Stern trawlers are restricted to depths of 50–100 metres, whereas pair trawlers operate in 18–50 metres. However, enforcement of these regulations remains weak due to limited resources within the Department of Fisheries. In recent years, an increase in industrial trawling effort has been observed, which has been associated with declines in demersal fish species, including catfish (Clarias gariepinus) [3–7].

Catfish (Clarias gariepinus) is an important commercial species in Africa, contributing significantly to trade, employment, and food security [8–11]. In Malawi, the species locally known as *mlamba* has gained increased importance following the decline of higher value species such as *chambo* (*Oreochromis spp.*) [12]. Demand remains high, particularly among high income households [13], and international demand has also increased. For instance, Samala and Kapute (2019) reported that catfish was the second most exported fish species from Malawi [14]. The species is found in several water bodies, including Lake Malawi, where it is harvested using gill nets in artisanal fisheries and stern or pair trawlers in commercial fisheries. Although artisanal fisheries contribute more total landings overall, trawlers yield significantly higher catches of catfish compared to gill nets [3].

The Gordon–Schaefer (GS) model, originally developed as a static equilibrium bioeconomic model, integrates biological and economic aspects of fisheries. It was later extended by Clarke and Munro into a dynamic framework, with further developments leading to nonlinear and non-autonomous formulations [15]. The model is widely used to evaluate fishery performance through reference points that reflect biological and economic objectives.

Key reference points include maximum sustainable yield (MSY), maximum economic yield (MEY), and open-access yield (OAY). MSY represents the highest catch that can be sustainably harvested, focusing primarily on biological conservation [16,17]. However, it does not account for economic efficiency. MEY addresses this limitation by incorporating profit maximisation alongside biological sustainability, representing the socially optimal harvest level [18,19]. In contrast, OAY represents an unregulated fishery condition where effort is not controlled. These three reference points are static in nature. A dynamic extension is the optimum sustainable yield (OSY), which incorporates discounting effects on biomass, effort, and yield [15].

Surplus production models such as the GS model are particularly useful in data-poor fisheries where time series of catch and effort data are the primary available information [20]. This is because more data-intensive approaches, such as age-based, length-based, or yield-per-recruit models, are often not feasible [21,22]. In Malawi, limitations in data availability and accuracy further restrict the application of such advanced methods [5,23].

Previous studies in Malawi have applied the GS model to fisheries such as *chambo* (*Oreochromis spp.*) and *usipa* (*Engraulicypris sardella*), revealing evidence of overexploitation [6,24–26]. However, these studies largely relied on static formulations of the model and focused on MSY, MEY, and OAY, without incorporating dynamic optimisation through OSY. In addition, bifurcation analysis of fishing effort was not explored, limiting understanding of critical thresholds in fishery dynamics.

More recently, a GS model application to the catfish fishery in the southeast arm of Lake Malawi reported stock depletion [27]. However, that study did not identify optimal levels of fishing effort or determine thresholds necessary to prevent further depletion and support recovery. Consequently, despite the economic and ecological importance of catfish, there remains limited information on optimal exploitation strategies for the fishery.

This study therefore aims to estimate the optimal fishing effort that maximises net revenue while maintaining biomass at sustainable levels in the commercial catfish (Clarias gariepinus) fishery of the southeast arm of Lake Malawi. Using empirical data and the GS framework, the study evaluates MSY, MEY, and OAY and conducts bifurcation analysis to examine equilibrium stability and identify critical effort thresholds. Beyond the equilibrium framework, the GS model is extended to its dynamic form to estimate OSY-based optimal biomass, yield, fishing effort, and net revenue under alternative discount rates. Overall, the study contributes to fisheries management by integrating equilibrium bioeconomic analysis, dynamic optimisation, and bifurcation analysis to identify sustainable and economically efficient exploitation strategies for the catfish fishery in the southeast arm of Lake Malawi.

## Materials and methods

### Study area

The study was conducted in the southeast arm of Lake Malawi, which covers an area of approximately 2,000 km^2^ [28]. The analysis included all six fisheries strata within the southeast arm, namely: 2.1 (Southwest Boadzulu), 2.2 (Southeast Boadzulu), 2.3 (Northwest Boadzulu), 2.4 (Northeast Boadzulu), 2.5 (Makanjira), and 2.6 (Fort Maguire).

### Data sources

The data used in this study were obtained from a published study on the commercial catfish fishery in the southeast arm of Lake Malawi [27]. Time-series catch and effort data for the commercial catfish (Clarias gariepinus) fishery covering the period 2000–2023 were sourced from the Traditional Fisheries Database maintained at the Fisheries Research Station of the Department of Fisheries in Monkey Bay, Mangochi District. Effort was measured as the annual number of fishing trips undertaken by commercial fishers, while catch represented aggregated annual landings, measured in tonnes, from all six strata of the southeast arm of Lake Malawi.

Price and cost data reported in [27] were collected through a field survey conducted between June and December 2023 involving 142 commercial fishers from the southeast arm of Lake Malawi. Participants were identified using a snowball sampling technique, which facilitated access to experienced commercial fishers [26,27]. This approach was adopted because a complete sampling frame of commercial fishers was not available, making random sampling impractical. According to [27], ethical considerations were observed throughout the study, including obtaining informed consent from all participants and ensuring confidentiality of the information collected.

Due to the unavailability of historical price and cost data, average estimates for 2023 were used in the analysis. The price variable represented the average market price per tonne of catfish (Clarias gariepinus) in 2023. Total fishing cost was estimated as the sum of fixed, variable, and opportunity costs incurred during the same period. Fixed costs included trawler acquisition, licence fees, and depreciation, while variable costs comprised operational expenses such as fuel, food, and crew labour. Opportunity costs represented forgone income from alternative economic activities. These cost components were incorporated in estimating the total cost of fishing [27].

### The Gordon-Schaefer model

The Gordon-Schaefer model is given by


$$\begin{array}{c} \frac{d}{dt}[x(t)]=rx(t)\left(1-\frac{x(t)}{K}\right)-qE(t)x(t),\;x(0)=x_{0} \end{array}$$ (1)

where *x*(*t*) is the stock biomass or the size of fish population, *x*_0_ is the initial population size, *r* is the growth rate of the fish biomass, *q* is the catchability coefficient, *E* is the effort, and *K* is the carrying capacity, which is the maximum number of fish that can be supported by the environment without causing any harm to the fish population [17,29,30]. The harvest or yield is given by


$$\begin{array}{c} H(t)=qE(t)x(t). \end{array}$$ (2)

Catch per unit effort (CPUE) is a, typically annual, measure of the amount of fish caught per unit of fishing effort. The following is the formula for CPUE


$$\begin{array}{c} CPUE=\frac{H}{E}=qK-\frac{q^{2}KE}{r}. \end{array}$$ (3)

CPUE is used to indicate fish stock abundance. Higher CPUE values indicate a healthy fish population, while decreasing CPUE values indicate overexploitation or decline in fish stock or biomass [6].

The GS model assumes logistic population growth, that growth rate is highest when the fishery population is small, a closed population, thereby ignoring species interactions such as competition and predation [31]. It further assumes that both biological parameters, namely the intrinsic growth rate, carrying capacity, and catchability coefficient, and economic parameters, including price and cost, remain constant over time [32]. Additional assumptions include that catch per unit of effort (CPUE) is proportional to biomass, there is no immigration or emigration, gear efficiency is constant, environmental variability does not affect the fish population, and fishing and natural mortality occur simultaneously [18]. Although many of these assumptions may not be met in practice, if used critically, the GS model is a powerful tool for stock assessment [17].

An **equilibrium** (or equilibrium point) of a dynamical system generated by an autonomous ordinary differential equation is a solution that does not change with time [33]. There are two equilibrium points, also called fixed points, associated with Equation 1. These are 0 and a positive fixed point


$$\begin{array}{c} x_{eqm}=K\left(1-\frac{qE}{r}\right) \end{array}$$ (4)

provided that $E<\frac{r}{q}$. When $E\geq \frac{r}{q}$, $x_{eqm}\leq 0$, and the population goes into extinction [15].

Bifurcation for a dynamical system, like the one in Equation 1, is a qualitative change in terms of structure and behaviour of the solutions to the system as a parameter is changed. A system may become fixed, settle down to a state of equilibrium or fluctuate chaotically. Bifurcation point is a point where this change takes place. The bifurcation point for the system in 1 is given by $E=\frac{r}{q}$ [17].

### Parameter estimation

Biological parameters (*r*, *q*, and *K*) were estimated by fitting a dynamic Schaefer surplus-production model to annual catch and effort data in R (version 4.5.2) and RStudio (version 2025.09.2−418). Biomass dynamics were simulated using a forward-Euler approximation of the surplus-production equation with annual time steps to match the temporal resolution of the data.

Model parameters were estimated by minimizing the sum of squared errors between observed and predicted catches on the logarithmic scale, assuming lognormally distributed observation errors. Optimisation was performed using the L-BFGS-B algorithm with bounded parameter constraints.

This modelling framework follows the general surplus-production modelling approach implemented in fisheries assessment software such as the TropFishR package [34], which includes tools for fitting Schaefer-type production models to catch–effort time-series data. Although TropFishR is primarily designed for fisheries stock assessment, particularly length-based and length-frequency analyses, it also provides functionality for surplus-production modelling [35]. However, the present study employed a custom implementation of the dynamic Schaefer model to allow explicit specification of the objective function, numerical integration scheme, parameter constraints, and uncertainty estimation via parametric bootstrap.

Parameter uncertainty was quantified using a parametric bootstrap with 1,000 replicates under lognormal observation error assumptions. Ninety-five percent confidence intervals were derived from the empirical distribution of bootstrap estimates. Non-convergent optimisation runs or biologically infeasible parameter sets resulting in negative biomass trajectories were excluded. Effort data were scaled prior to estimation to improve numerical stability.

### Reference points

The Maximum Sustainable Yield (MSY) is the greatest amount of fish that can be harvested while keeping the fish stock sustainable. A catch level is sustainable if it does not exceed the growth rate since it can be maintained forever. According to [17], for the GS model, stock amount at MSY is


$$\begin{array}{c} x_{MSY}=\frac{K}{2}, \end{array}$$ (5)

sustainable catch is


$$\begin{array}{c} h_{MSY}=\frac{rK}{4} \end{array}$$ (6)

and effort which gives sustainable yield is given by


$$\begin{array}{c} E_{MSY}=\frac{r}{2q}. \end{array}$$ (7)

MSY is a biological management indicator and including economic parameters in the model 1 gives bioeconomic reference points. The net revenue is the difference between total revenue TR=price(p)×catch(h) and total cost TC=unit cost(c)×effort(E):


$$\begin{array}{c} \text{sustainable net revenue}\;(\pi )=ph-cE=pqEK\left(1-\frac{qE}{r}\right)-cE. \end{array}$$ (8)

Maximum Economic Yield (MEY) is the yield level which maximises sustainable net return from the fishing activities [36]. MEY is the preferred reference point since it is used to maximise long term profits which requires conserving the fishery biologically [31]. The biomass level at MEY is given by


$$\begin{array}{c} x_{MEY}=\frac{K}{2}\left(1+\frac{c}{pqK}\right), \end{array}$$ (9)

effort, which maximises net returns, is given by


$$\begin{array}{c} E_{MEY}=\frac{r}{2q}\left(1-\frac{c}{pqK}\right) \end{array}$$ (10)

and catch at MEY is


$$\begin{array}{c} h_{MEY}=\frac{rK}{4}\left(1-{\left(\frac{c}{pqK}\right)}^{2}\right). \end{array}$$ (11)

In Equations 8–11, *r*, *q* and *K* are biological parameters, namely: intrinsic growth rate, catchability coefficient, and carrying capacity, respectively. Additionally, *p* and *c* are economic parameters representing price and cost, respectively.

Open access yield (OAY) is the level of fishing where there are no restrictions on gear types, number of fishers or the amount of fishing effort [18]. Any interested individual can take part in OAY and this often results into overfishing. This is obviously not sustainable and can lead into extinction of the fishery resources. The biomass level at OAY is given by


$$\begin{array}{c} x_{OAY}=\frac{c}{pq}, \end{array}$$ (12)

while the effort at OAY is


$$\begin{array}{c} E_{OAY}=\frac{r}{q}\left(1-\frac{c}{pqK}\right) \end{array}$$ (13)

provided that *pqK* > *c*. OAY harvest level is given by


$$\begin{array}{c} h_{OAY}=\frac{rc}{pq}\left(1-\frac{c}{pqK}\right). \end{array}$$ (14)

The three reference points, MSY, MEY and OAY, are static in nature. Optimum Sustainable Yield (OSY) is the only dynamic reference point for the GS model. OSY looks at the effects of discounting on biomass, effort and yield. According to [36], the optimal biomass amount is given by the equation


$$\begin{array}{c} x^{*}=\frac{K}{4}\left[\left(1+\frac{c}{pqK}-\frac{\delta }{r}\right)+\sqrt{{\left(1+\frac{c}{pqK}-\frac{\delta }{r}\right)}^{2}+\frac{8\delta c}{pqrK}}\right] \end{array}$$ (15)

where δ is discount rate. The fishing effort is


$$\begin{array}{c} E^{*}=\frac{r}{q}\left(1-\frac{x^{*}}{K}\right), \end{array}$$ (16)

while the harvest or yield level is


$$\begin{array}{c} h^{*}=qE^{*}x^{*}. \end{array}$$ (17)

The following equation gives the optimal annual sustainable net revenue


$$\begin{array}{c} \pi =ph^{*}-cE^{*}. \end{array}$$ (18)

### Sensitivity analysis

The study used historical catch and effort data for the commercial catfish fishery in the southeast arm of Lake Malawi covering a 24-year period from 2000 to 2023. However, historical economic data on fish price and fishing cost were unavailable. Consequently, the study adopted the Gordon–Schaefer model assumption of constant price and cost and used average price and cost estimates for 2023 in the analysis. In practice, however, economic conditions may vary substantially over time, and such variations may influence derived model outputs, including optimal fishing effort estimates. To assess the robustness of the estimated effort reference points to changes in economic conditions, sensitivity analysis was conducted on the price and cost parameters.

Sensitivity analysis was performed by varying the baseline price and cost values independently by ±30% in intervals of 5%, while holding all other model parameters, namely the intrinsic growth rate (*r*), catchability coefficient (*q*), and carrying capacity (*K*), constant. For each variation in price and cost, the corresponding fishing effort levels at maximum economic yield (MEY) and open-access yield (OAY) were recalculated using the Gordon–Schaefer model equations. The resulting effort estimates were then plotted to evaluate the sensitivity of optimal fishing effort to changes in economic parameters. The analysis was implemented using Python (version 3.13) with custom scripts.

## Results

### Catch, effort and catch per unit effort (CPUE)

Table 1 shows time series of catch and effort for commercial catfish (Clarias gariepinus) from the southeast arm of Lake Malawi during the study period (2000–2023). During this period total catch of commercial catfish (Clarias gariepinus) was 2071.2 tonnes and the average catch was 86.3 tonnes. The maximum catch of 149.8 tonnes was observed in 2000 and the minimum catch of 20.2 tonnes was observed in 2019. Although the catch levels have fluctuated over the study period, the declining trend is still observable. For example, the harvest or yield is below 100 tonnes for 16 years out of 24 years under study. This is from 2004 to 2007, from 2010 to 2015, and from 2018 to 2023. The total and average effort values were 53064 and 2211 trips respectively. The highest effort of 4738 trips was recorded in 2016 and the lowest effort of 1207 trips was observed in 2023.

**Table 1:** Catch trend for commercial catfish (Clarias gariepinus) fishery.

Year	Catch (tonnes)	Effort (Trips)	CPUE
2000	149.8	3420	0.043800
2001	141.3	2874	0.049155
2002	119.7	1285	0.093136
2003	122.5	1691	0.072414
2004	94.4	1702	0.055485
2005	55.2	1361	0.040573
2006	89.2	1492	0.059817
2007	86.0	1434	0.059946
2008	119.1	2027	0.058764
2009	112.7	2594	0.043435
2010	91.0	2380	0.038229
2011	62.1	1432	0.043383
2012	49.2	1569	0.031366
2013	78.1	2646	0.029520
2014	75.3	2076	0.036276
2015	67.5	1635	0.041323
2016	113.0	4738	0.023842
2017	132.1	3331	0.039643
2018	93.7	3717	0.025200
2019	20.2	2902	0.006957
2020	32.9	2057	0.015988
2021	48.9	1962	0.024931
2022	67.5	1532	0.044091
2023	49.8	1207	0.041245

Catch and effort were measured in tonnes and number of fishing trips, respectively. CPUE (catch per unit effort) represents the relationship between catch and fishing effort. CPUE values are reported in tonnes per trip. Because the values are small, they may be interpreted more easily by multiplying them by 100.

**Fig 1:**
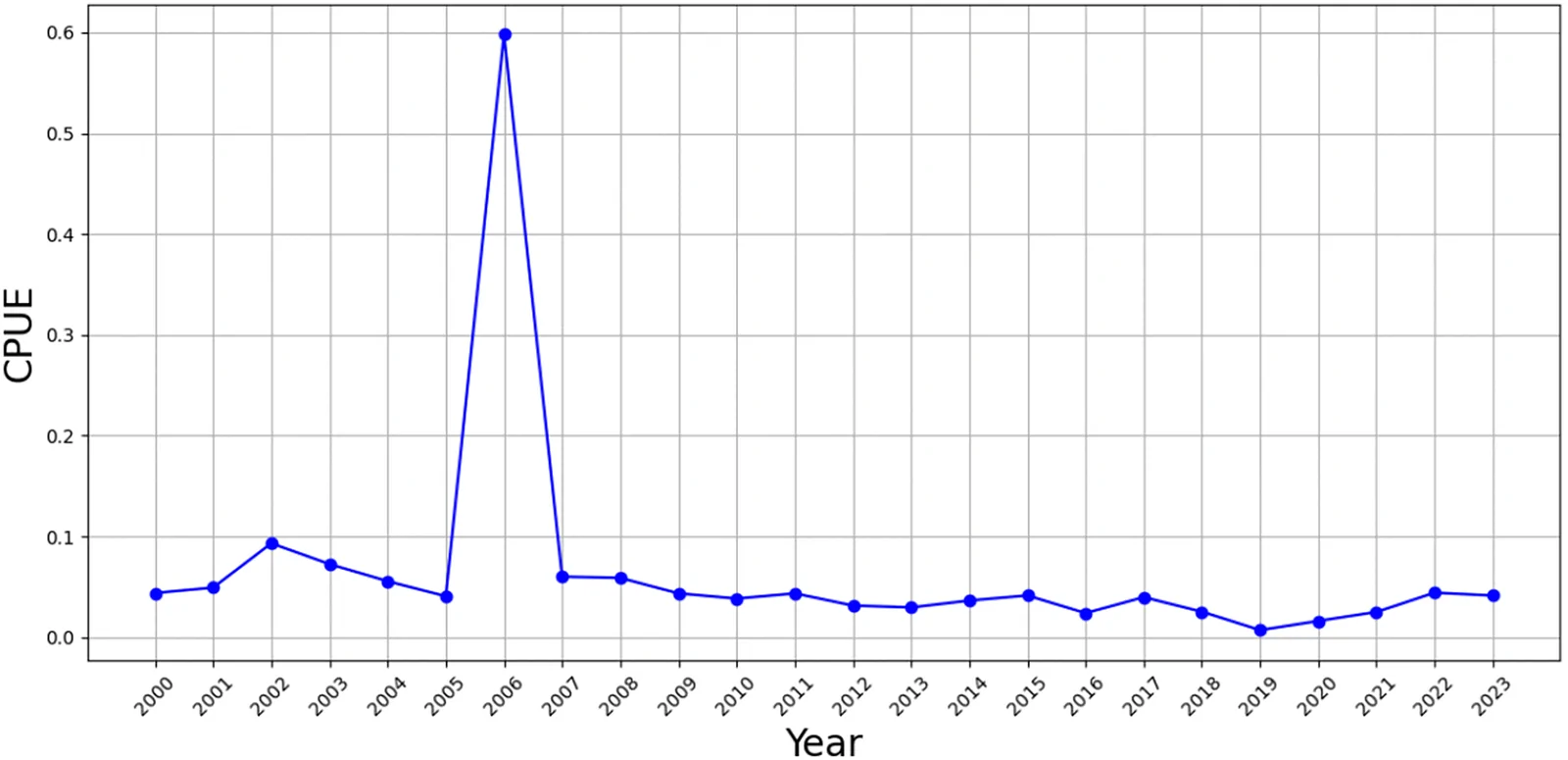
Catch per unit effort (CPUE) trend over time (tonnes per trip). The figure shows a general declining trend in CPUE, indicating increasing difficulty in catching fish for the same level of fishing effort over time.

Table 1 also shows catch per unit of effort (CPUE) for commercial catfish (Clarias gariepinus) from southeast arm of Lake Malawi between 2000 and 2023. The highest CPUE of 0.093136 was recorded in 2002 and the lowest CPUE of 0.006957 was observed in 2019. The average CPUE throughout the period under study was 0.042438. Fig 1 is the graph of CPUE for commercial catfish (Clarias gariepinus) fishery from southeast arm of Lake Malawi. The graph shows a general decline in CPUE over time.

### Estimated biological and economic parameters

Biological parameters were estimated by fitting the Schaefer model to the data presented in Table 1 using R (version 4.5.2; [37]) within RStudio (version 2025.09.2−418). The estimated parameters, *r*, *q*, and *K*, along with their bootstrap 95% confidence intervals, are shown in Table 2.

**Table 2:** Biological parameters for the model.

Parameter	Description	Value	95% CI	Unit
*r*	Intrinsic growth rate	0.683	(0.292, 2.000)	year^-1^
*q*	Catchability coefficient	0.0001	(0.0000518, 0.0001)	trip${\;}^{-1}{\text{year}}^{-1}$
*K*	Carrying capacity	557.4	(378.1, 1498.0)	tonne

The intrinsic growth rate (*r*) measures population growth per year, catchability coefficient (*q*) measures number of trips per yaer, and carrying capacity (*K*) is measured in tonnes. All parameters are estimated with corresponding 95% confidence intervals.

The economic data used in this study are based on the same dataset employed by [27] for the commercial pair-trawl fishery in the southeast arm of Lake Malawi. While [27] reported only the final economic parameters used in the bioeconomic analysis, the present study provides additional detail on the underlying cost components, assumptions, and calculations used to derive the fishing cost and fish price parameters. A representative pair trawler was used as the unit of analysis, with fishing effort measured in trips per year. The analysis is formulated within the standard Gordon–Schaefer framework, in which effort is defined as fishing trips per vessel per year, and the model does not explicitly represent fleet size, entry, or exit decisions. In this specification, all annual fishery costs (fixed, variable, and opportunity costs) are aggregated and expressed as a single constant cost-per-unit-effort parameter, consistent with the standard Gordon–Schaefer formulation. The annual fixed costs were used only in the derivation of this average cost parameter and are not represented as a separate fixed-cost term in the optimization model. Consequently, the model assumes a constant average cost of effort and does not distinguish between fixed and variable cost components during optimization. Accordingly, the model should be interpreted as a stylised representation of fishing effort rather than a vessel-level accounting model. Effort therefore captures variation in fishing intensity rather than changes in the number of vessels or licences.

Based on the fishing calendar and operational characteristics described in [27], supplemented by the authors’ field knowledge of the fishery, fishing activity occurs approximately 3 days per week (about 12 days per month), subject to interruptions from adverse weather and the annual closed season from 1 November to 31 January. This corresponds to an effective fishing period of approximately 9 months per year, or about 108 fishing trips per vessel annually.

Total annual cost consists of fixed, variable, and opportunity costs. Fixed costs include vessel depreciation, licence and registration fees, and vessel purchase costs, while variable costs include fuel, food, and labour expenses. Opportunity cost was approximated using the 2023 minimum wage as an estimate of alternative labour income (MK 38 000 per month, giving a total of MK 456 000 per year). Fuel consumption was assumed to be 100 litres per trip at a fuel price of MK 2 734 per litre (2023). This resulted in an annual fuel cost of MK 29 527 200. A fishing trip was assumed to involve an average of eight crew members, each spending about MK 500 on food per trip. Therefore, the total annual food cost was MK 432 000. For labour, each crew member receives an average payment of MK 3 000 per trip, giving a total annual labour cost of MK 2 592 000. Using the estimated annual cost, the average fishing cost was calculated as MK 351 548 per trip by dividing the total annual cost (MK 37 967 200) by 108 trips per year. The landing price used in the analysis was MK 7 000 per kilogram, equivalent to MK 7 000 000 per tonne, based on 2023 market averages. The cost components, assumptions, and economic parameters used in the model are summarised in Table 3.

**Table 3:** Economic data for the fishery.

Item	Description	Amount
	Purchase value of pair trawler	MK 4 000 000
Fixed costs	Depreciation (at 4%)	MK 160 000
	Licence/registration	MK 800 000
**Total fixed cost**		**MK 4 960 000**
	Fuel	MK 29 527 200
Variable costs	Food	MK 432 000
	Labour	MK 2 592 000
**Total variable cost per year**		**MK 32 551 200**
Opportunity cost	Minimum wage of labour per month	MK 38 000
**Total opportunity cost per year**		**MK 456 000**
Total cost per year		MK 37 967 200
**Cost per fishing trip**		**MK 351 548**
	Price per kg	MK 7 000
Landing price	Price per tonne	**MK 7 000 000**

The estimated economic parameters are the fishing cost (c = 351 548 MK per trip) and the fish price (p = 7 000 000 MK per tonne). The cost parameter represents the average cost per fishing trip per vessel, obtained by dividing total annual cost by 108 trips per year. The price parameter is expressed per tonne of fish landed.

### MSY, MEY, OAY and sustainable net revenue

Table 4 shows stock size (*x*), effort (*E*), harvest (*h*), and sustainable net revenue at MSY, MEY and OAY reference points. Equations 5, 9 and 12 were used to calculate stock size or biomass at MSY, MEY and OAY, respectively. Equations 7, 10 and 13 were used to calculate effort at MSY, MEY and OAY, respectively. For yield or harvest at MSY, MEY and OAY, equations 6, 11 and 14 were used, respectively. The sustainable net revenue was computed by substituting effort and harvest values at MSY and MEY into Equation 8.

**Table 4:** Stock size, effort and harvest at MSY, MEY and OAY.

Exploitation level	Stock size (*x*)	Effort (*E*)	Harvest (*h*)	S. Net revenue (MK yr^-1^)
MSY	288.70	3415.00	98.59	−510399070.00 ($ −291 490.05)
MEY	539.81	444.70	24.01	11 702 688.35 ($ 6 683.43)
OAY	502.21	889.40	44.67	0.00

Stock size (*x*) represents the estimated population size measured in tonnes, effort (*E*) is measured in fishing trips per year, and harvest (*h*) denotes the total catch in tonnes per year. Sustainable net revenue is defined as the difference between total revenue and total costs. The negative sustainable net revenue estimated at MSY may reflect depleted stock conditions in the fishery, as reported by [27]. Values in brackets represent the USD equivalents, converted using the 2023 exchange rate of MK1 751 per USD.

The MEY reference point gives highest stock size (539.81 tonnes), highest sustainable net revenue (11 702 688.35 Malawi Kwacha), lowest effort (about 445 trips) and lowest harvest (24.01 tonnes). The MSY reference point gives highest fishing effort and negative sustainable net revenue. The OAY gives zero sustainable net revenue.

### Bifurcation analysis

The two fixed points for the GS model 1 are 0 and $x_{eqm}=K\left(1-\frac{qE}{r}\right)$, and the bifurcation point is $E=\frac{r}{q}$. Using values in Table 2, the positive fixed point is 288.7, and the bifurcation point is *E* = 6830.

**Fig 2:**
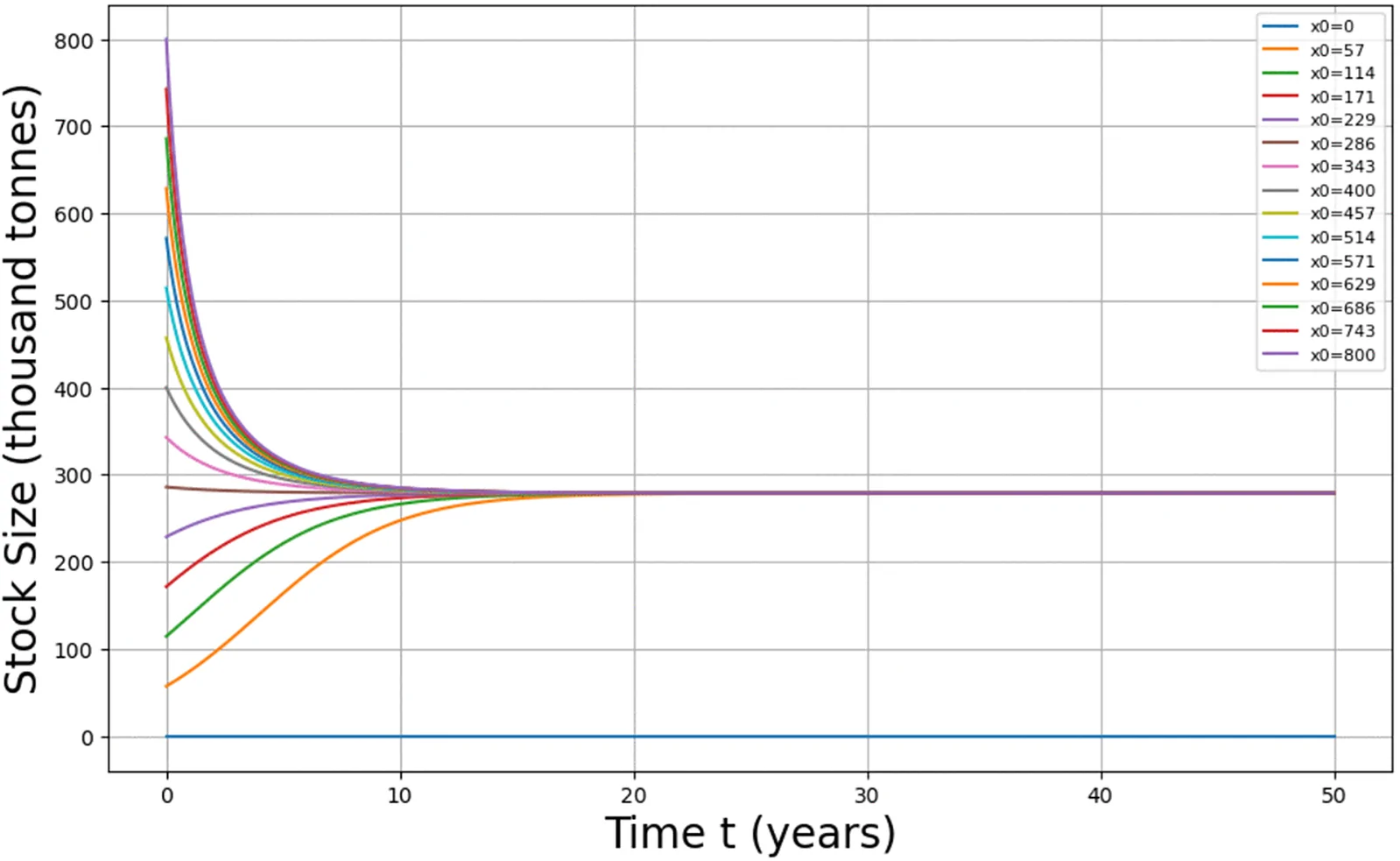
Solution curves for the GS model when fishing effort is *E* = 3415. This shows population trajectories given different initial populations.

Fig 2 shows solution curves for $E=E_{MSY}=3415$. Two values of fixed points, 0 and 288.7, exist. If the initial population starts at any of the two fixed points, it remains there. However, for any initial stock size greater than 288.7, the population declines to equilibrium point 288.7. Additionally, for initial population of between 0 and 288.7 the population grows up to 288.7, in the long run. This means the fixed point 0 is unstable, while the fixed point $x_{MSY}=288.7$ is stable. Therefore, applying fishing effort of 3415 trips ensures existence of catfish (Clarias gariepinus) stocks of size half the carrying capacity.

**Fig 3:**
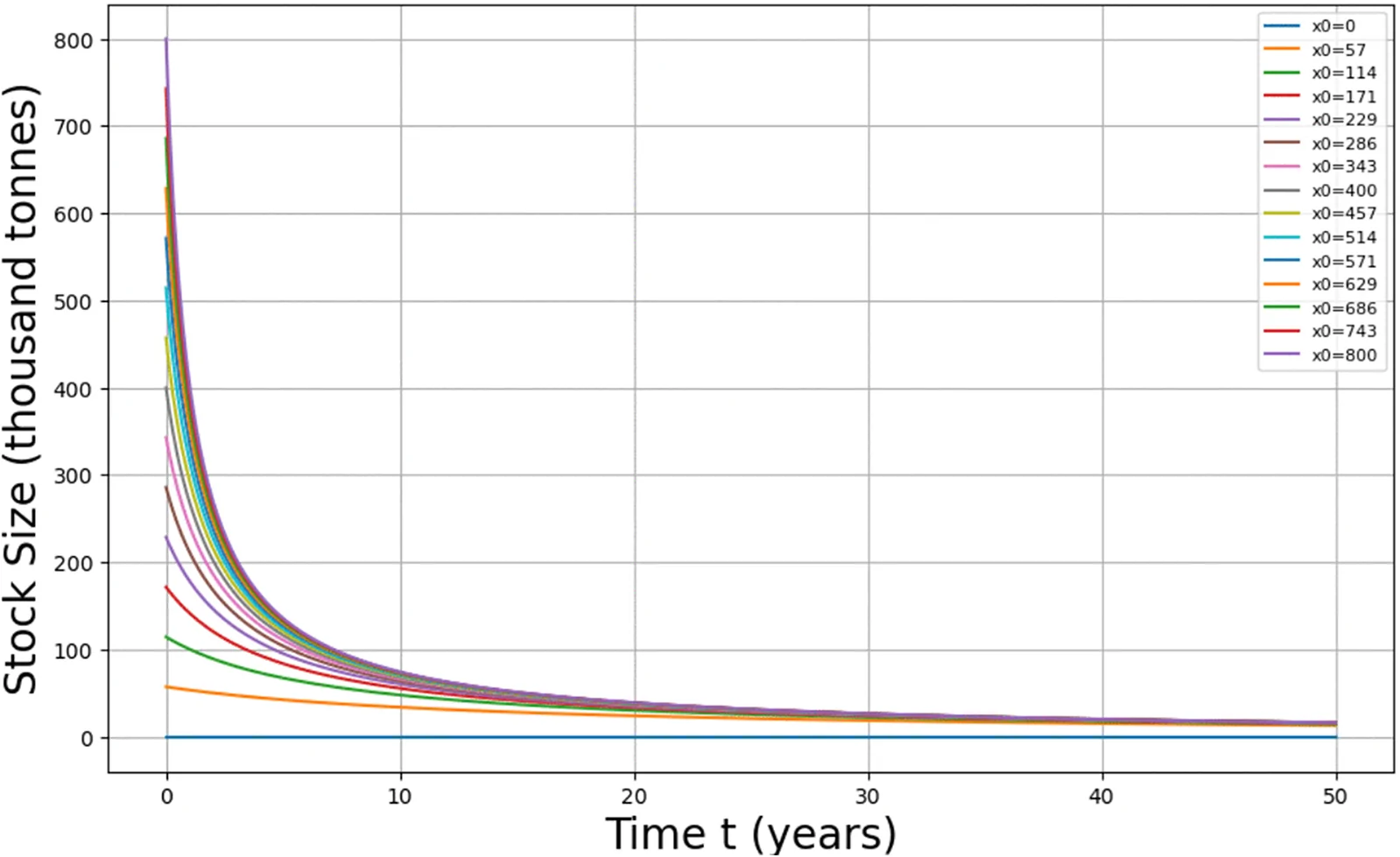
Solution curves for *E* = 6830. This shows population trajectories given different initial populations when fishing effort is 6830 trips per year.

Fig 3 presents solution curves for the case where *E* = 6830 trips per year which is the bifurcation point. There is only one fixed point which is 0. If the initial stock size is 0, it remains there forever. However, if the initial stock size is greater than 0, with passage of time, the population declines to a point where all fish are gone. This means 0 is a stable fixed point. Therefore, applying effort of 6830 trips leads to extinction of the catfish (Clarias gariepinus) from the southeast arm of Lake Malawi.

**Fig 4:**
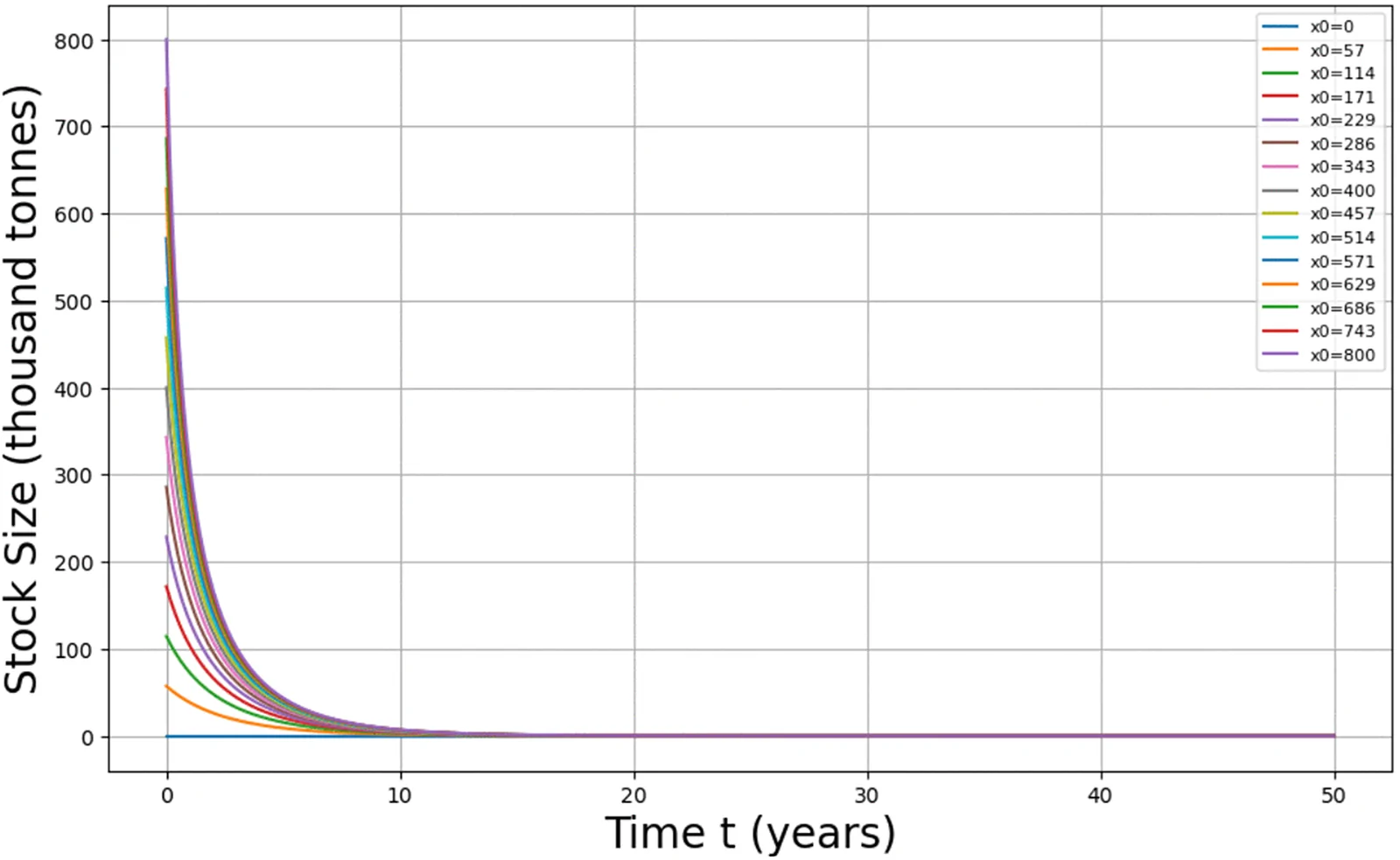
Solution curves for *E* = 10245. This shows population trajectories given different initial populations when fishing effort is 10245 trips per year.

Fig 4 shows solution curves for *E* = 10245 trips per year, which is greater than the bifurcation point. There is one fixed point 0. If the initial stock size is 0, it remains there indefinitely. For any initial stock size of greater than 0, the population goes down to 0. This means 0 is a stable fixed point, and the catfish (Clarias gariepinus) population goes into extinction regardless of initial amount of fish.

### The OSY reference point

Table 5 presents stock size ($x^{*}$), effort ($E^{*}$), harvest ($h^{*}$), and sustainable net revenue at the dynamic reference point of the Gordon–Schaefer model, the optimal sustainable yield (OSY). At a zero discount rate (δ=0), the OSY collapses to the maximum economic yield (MEY), such that $x^{*}$, $E^{*}$, and $h^{*}$ are identical to $x_{MEY}$, $E_{MEY}$, and $h_{MEY}$, respectively. As the discount rate increases, effort and harvest increase, while stock size and sustainable net revenue decrease. Conversely, lower discount rates are associated with lower effort and harvest and higher stock biomass. These results show that the OSY solution changes consistently with the discount rate, where higher discounting leads to increased exploitation and reduced stock levels within the model framework.

**Table 5:** Stock size, effort, harvest and sustainable net revenue at OSY.

δ	Stock size ($x^{*}$)	Effort ($E^{*}$)	Harvest ($h^{*}$)	S. Net revenue (MK yr^-1^)
0	539.81	444.70	24.01	11 702 688.35
0.05	538.39	461.50	24.85	11 685 991.06
0.10	537.06	477.14	25.63	11 640 389.14
0.20	534.67	505.41	27.02	11 484 556.44
0.30	532.58	530.22	28.24	11 269 872.21
0.40	530.72	552.14	29.30	11 019 548.47
0.50	529.07	571.63	30.24	10 749 191.03
0.60	527.60	589.07	31.08	10 469 309.56
0.70	526.28	604.74	31.83	10 186 967.25
0.80	525.08	618.90	32.50	9 906 877.39
0.90	523.99	631.75	33.10	9 632 140.15
0.95	523.49	637.74	33.38	9 497 433.09
∞	502.21	889.40	44.67	0.00

Stock size ($x^{*}$), effort ($E^{*}$), harvest ($h^{*}$), and sustainable net revenue are calculated at different discount rates (δ). For this fishery, moderate positive discount rates are considered more realistic, as they reflect a balance between short-term economic benefits and long-term stock sustainability.

Table 5 shows that as the discount rate becomes very large, the optimal biomass level approaches that of the open-access equilibrium (OAY). This implies that in the limiting case of infinite discounting, future benefits are completely disregarded, and the model converges to the OAY outcome:


$$\begin{array}{c} \lim_{\delta \to \infty }x^{*}=x_{OAY}. \end{array}$$

This result can be verified by simplifying the expression for $x^{*}$ in Equation 15. Algebraic manipulation of the optimal stock equation, followed by taking the limit as δ→∞, yields:


$$\begin{array}{c} \lim_{\delta \to \infty }x^{*}=\frac{c}{pq}=x_{OAY}. \end{array}$$

The detailed algebraic steps leading to this result have been omitted here for clarity but follow from standard limit evaluation techniques.

### Sensitivity of effort to price and cost

**Fig 5:**
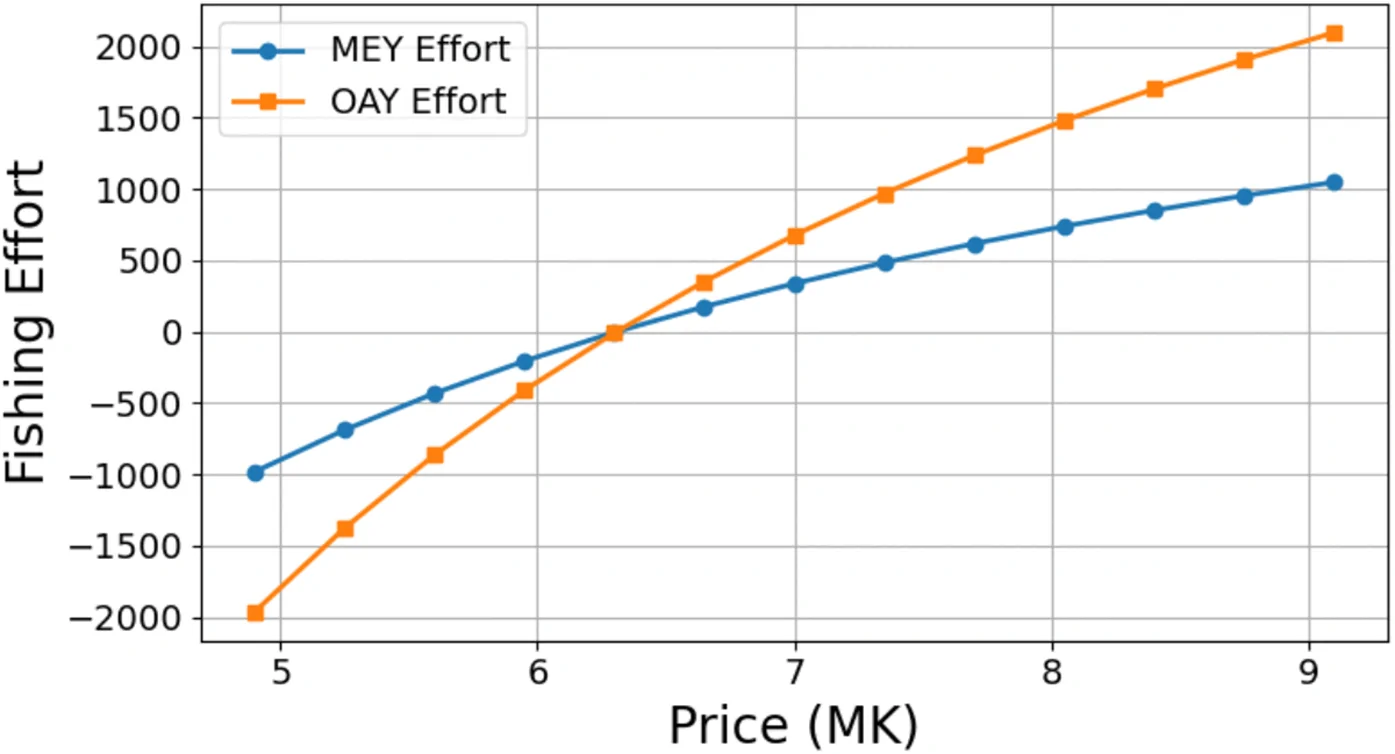
Sensitivity of fishing effort to price. This shows how fishing efort responds to changes in price.

Fig 5 presents the sensitivity of fishing effort at the maximum economic yield (MEY) and open access yield (OAY) reference points to changes in price, expressed in million Malawi Kwacha. The baseline price of MK 7 000 000 was increased and decreased by ±30% in intervals of 5%, while all other parameters, including cost, were held constant.

Fig 5 shows that fishing effort increases with increasing price at both the MEY and OAY reference points, indicating positive sensitivity to price changes. The steep increase in effort suggests a strong positive relationship between fishing effort and price. At price levels below the baseline value, effort at the MEY reference point is higher than at the OAY reference point. In contrast, at price levels above the baseline value, effort at the OAY reference point exceeds that at the MEY reference point, indicating that the OAY regime responds more strongly to increases in price.

**Fig 6:**
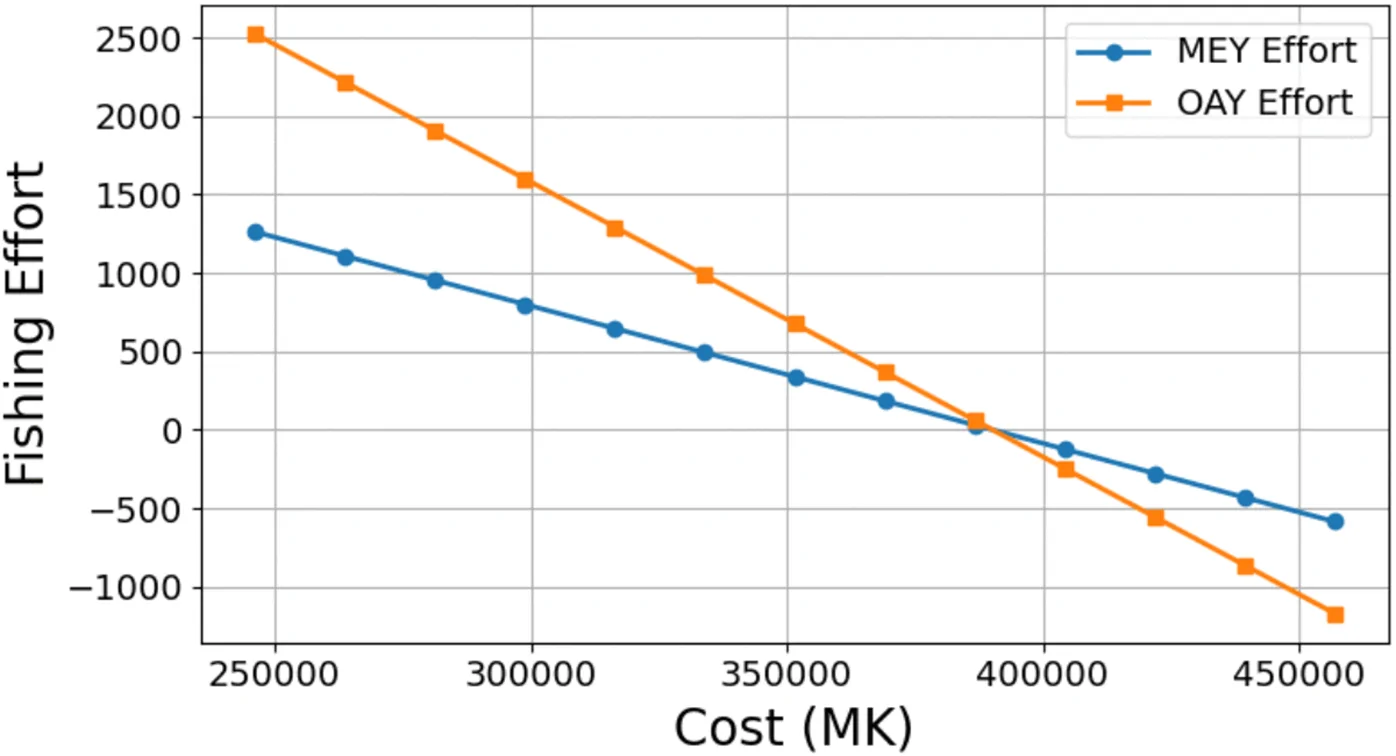
Sensitivity of fishing effort to cost. This shows how fishing efort responds to changes in cost.

Fig 6 presents the sensitivity of fishing effort at the MEY and OAY reference points to changes in cost. The baseline cost of MK 351 548 was increased and decreased by ±30% in intervals of 5%, while all other parameters, including price, were held constant.

Fig 6 shows that fishing effort at both the MEY and OAY reference points decreases as cost increases, indicating negative sensitivity to changes in cost. The steep decline in effort suggests a strong negative relationship between fishing effort and cost. At cost levels below the baseline value, effort at the OAY reference point is greater than effort at the MEY reference point. However, at cost levels above the baseline value, effort at the MEY reference point exceeds that at the OAY reference point, indicating that fishing effort under the OAY regime is more sensitive to increases in cost than effort under the MEY regime.

## Discussion

Declining CPUE (Fig 1) indicates that catfish became increasingly difficult to catch for the same level of fishing effort. This pattern suggests diminishing returns to effort, likely associated with increased fishing pressure and/or stock depletion. The trend is consistent with predictions of the Gordon–Schaefer model, which posits that increasing fishing effort eventually leads to reduced stock biomass and lower catch per unit effort [6,18,32]. This observation is also consistent with the findings of [27], which indicate that this fishery is already depleted. This has important implications for fishery management, as declining CPUE may signal that current effort levels are approaching or exceeding sustainable limits, potentially resulting in long-term economic and ecological losses if not addressed.

The low CPUE values observed in certain years, particularly 2019 and 2020, may reflect interannual variability in catch rates driven by changes in stock abundance, fishing conditions, and catchability. Such fluctuations may also be influenced by environmental variability and socio-economic factors affecting fishing activities during those years.

From Table 4, MEY is the reference point that gives highest biomass and maximum revenue. This means that, apart from providing highest economic returns, the MEY exploitation level also helps conserving fish stocks. These results are consistent with observations from several other studies in the field [15,24,38,39]. Note that all the effort values used in this study (Table 1) are greater than effort at MEY.

MSY yields the highest harvest among the three reference points (Table 4). However, the negative sustainable net revenue estimated at MSY indicates that operating at this level would result in financial losses, primarily due to high operational costs relative to revenues, and possibly low fish prices. This finding contrasts with most studies applying the Gordon–Schaefer model, which generally report positive sustainable net revenues [15,30,36,40]. Nevertheless, similar results were reported by Kar and Chakraborty [39], who demonstrated that fisheries may become economically unviable under certain conditions. In the present study, the negative net revenue at MSY may reflect specific characteristics of the fishery, including high fishing costs, low market prices, and depleted stock levels. Results reported by [27] indicate that the commercial catfish fishery in the southeast arm of Lake Malawi is already depleted, which may partly explain the economically unviable outcome at the MSY reference point. In addition, the use of 2023 economic data together with historical catch and effort data may have influenced the estimated net revenues, as current operational costs may not fully represent historical fishing conditions.

Note that the effort at MSY reference point is greater than the effort at OAY level. This means more effort is required in order to balance harvest rate with growth rate of the fishery, which is the objective at MSY. OAY gives zero sustainable net revenue since total revenue (TR) equals total cost (TC). This holds true when all significant figures of yield and effort are used in Equation 8. This outcome corroborates findings from other bioeconomic studies using the Gordon-Schaefer model [38,39].

Bifurcation analysis indicates that fishing effort levels below the bifurcation point support the persistence of catfish (Clarias gariepinus) in the southeast arm of Lake Malawi. The effort levels associated with the three reference points, namely *MSY*, *MEY*, and *OAY*, are all below the bifurcation threshold of 6830 trips/year. Similarly, all observed effort values reported in Table 1 remain below this threshold. According to the model, fishing at these effort levels would maintain a positive stock size and prevent stock collapse, thereby allowing the population to persist over time.

However, persistence of the stock does not necessarily imply that the biomass is healthy or maintained at optimal levels. Although all observed effort values in Table 1 are below the bifurcation threshold, the declining catch per unit effort (CPUE) suggests that the fishery may still be experiencing considerable fishing pressure and reduced stock abundance. In this context, the bifurcation point should be interpreted as a collapse threshold rather than an indicator of satisfactory stock condition. A fish population may persist below the bifurcation point while still undergoing gradual biomass decline due to factors such as changes in catchability, environmental variability, prolonged exploitation, and depletion of larger individuals within the stock.

These findings highlight the importance of maintaining fishing effort below the bifurcation point to avoid stock collapse. Nevertheless, remaining below this threshold alone may not be sufficient to ensure stock recovery or sustain high productivity. Consequently, fisheries management strategies should not only aim to keep effort below the bifurcation point, but also maintain fishing pressure at levels that promote biomass rebuilding and improved CPUE trends.

Results on the optimal sustainable yield (OSY) reference point suggest that as discount rate increases, effort and harvest increase while biomass and sustainable net revenue decrease. This is consistent with observations by Midan and Lee [41] who state that fishers prefer more current to future outcome. An increase in discount rate pushes fishers to catch more while decreasing discount rate makes fishers slow on catches and eventually leave the fish stock. Other researchers also made similar observations on the effects of increasing discount rate on effort, catch and sustainable net revenue [17,39].

The results also indicate that OSY converges with the maximum economic yield (MEY) reference point when the discount rate is zero, suggesting that in the absence of time preference, the fishery would be managed to maximise long-term economic benefits. However, as the discount rate increases, the OSY levels diverge from MEY, with higher effort and harvest levels leading to decreased stock size and net revenue. When the discount rate approaches infinity, the OSY biomass level converges to the open-access yield (OAY) level. These observations align with observations by Ibrahim [17] who states that with a high discount rate, future revenues do not count much. Instead, there is a tendency to maximise current revenues, leading to excessive harvesting, which goes down to $x_{OAY}$. However, prioritising short-term gains over long-term sustainability can have detrimental effects on the fishery’s ecological and economic health. The findings highlight the importance of considering discount rates in fisheries management, as they can significantly impact the trade-offs between economic and conservation objectives.

The study used catch and effort data spanning a 24-year period; however, historical economic data on fish prices and fishing costs were unavailable. Consequently, the analysis relied on economic price and cost data from a single year, 2023. This approach is consistent with one of the assumptions of the Gordon–Schaefer model, which assumes that price and cost remain constant over time. In reality, however, economic parameters in the fishery are likely to have varied substantially over the study period due to factors such as inflation, exchange rate fluctuations, changes in fuel prices, and variability in fish market prices. Such temporal variation could influence estimates of optimal fishing effort levels. Therefore, the use of single-year economic parameters represents an important limitation of the present study.

To evaluate the extent to which the estimates depend on the economic parameters, sensitivity analyses were conducted for both price and cost. The results showed that fishing effort at both the maximum economic yield (MEY) and open-access yield (OAY) reference points increased with higher prices and decreased with higher costs. These findings indicate that the estimated optimal effort levels are positively sensitive to price and negatively sensitive to cost. Economically, higher fish prices increase profitability and provide incentives for increased fishing effort, whereas higher fishing costs reduce profitability and discourage effort expansion. The analysis further demonstrated that even relatively modest changes in price and cost can produce noticeable changes in fishing effort, suggesting that variability in economic conditions may substantially influence bioeconomic targets in the fishery. Nevertheless, the overall trends and relationships predicted by the model remained consistent across the tested parameter ranges, indicating that the main conclusions of the study are reasonably robust despite uncertainty in the economic data. Furthermore, previous bioeconomic studies have also applied the assumption of constant economic parameters and relied on single-year economic data [6,24,27,31,32].

These findings highlight the importance of collecting historical economic data on fish prices and fishing costs alongside time-series catch and effort data. Such information would improve future bioeconomic assessments of the catfish fishery in the Southeast Arm of Lake Malawi. Incorporating time-varying economic parameters into future modelling efforts would likely provide more accurate and dynamic estimates of optimal fishing effort levels.

Price and cost data used in this study were derived from a field survey reported in [27], in which commercial fishers were recruited using snowball sampling. This approach facilitated the identification of experienced and active fishers operating in the southeast arm of Lake Malawi. However, snowball sampling is a non-probability sampling method and may introduce selection bias. In particular, fishers recruited through existing networks may share similar fishing practices, operational characteristics, or economic conditions, potentially resulting in the overrepresentation of certain groups while underrepresenting others. Consequently, the estimated economic parameters may not fully reflect the diversity of fishing operations within the fishery.

This limitation could influence the estimated optimal effort levels derived from the bioeconomic model. Nevertheless, in the absence of historical economic datasets for the fishery, the collected price and cost data provide important baseline estimates for assessing the fishery. Furthermore, the sensitivity analyses conducted on the price and cost parameters demonstrated that, although variations in economic parameters affected the magnitude of the estimated effort levels, the overall model trends and conclusions remained consistent across the tested parameter ranges.

Future studies should consider the application of probability-based sampling techniques and the integration of additional data sources, such as fisheries records, market surveys, and government statistics, to improve the representativeness and validation of economic parameter estimates.

Although the bioeconomic Gordon–Schaefer (GS) model provided useful estimates of optimal fishing effort, several of its assumptions may not fully hold for the commercial catfish fishery in the Southeast Arm of Lake Malawi. For example, the assumption of no immigration or emigration may be unrealistic because catfish may move between different parts of the lake, thereby affecting stock availability and catch rates in the Southeast Arm of Lake Malawi. In addition, the assumption of constant catchability may not hold because catchability can vary over time due to changes in fishing practices, fishing technology, gear efficiency, and fisher behaviour, all of which may alter the relationship between fishing effort and catch. If catchability increases while being assumed constant, the model may underestimate stock depletion and overestimate optimal fishing effort levels.

The model also assumes that environmental variability does not affect stock dynamics. However, changes in environmental conditions, such as temperature fluctuations, lake levels, increased turbidity, and low dissolved oxygen concentrations, may influence fish recruitment, growth, mortality, distribution, and behaviour. Such changes may reduce fish accessibility to commercial fishers by driving fish away from traditional fishing grounds, thereby affecting catch rates. Consequently, ignoring environmental variability may introduce uncertainty into estimates of intrinsic growth rate, catchability coefficient, and optimal fishing effort levels.

Despite these limitations, the GS model remains an important tool for conducting preliminary bioeconomic assessments, particularly in data-limited fisheries. The model enables evaluation of broad bioeconomic trends and management reference points using the available data. Nevertheless, the preceding discussion highlights the importance of interpreting the model results with caution. Future studies should incorporate environmental variability, time-varying catchability, and spatial stock dynamics to improve the reliability of stock and bioeconomic assessments.

### Management implications

The results of this study indicate that the MSY reference point is economically unviable, as it is associated with negative sustainable net revenue. This suggests that operating at MSY may result in financial losses for fishers. Therefore, fisheries management authorities should consider excluding MSY as a target reference point in this fishery.

In contrast, the MEY reference point provides optimal outcomes in terms of both economic performance and biological sustainability. At MEY, sustainable net revenue is maximised, and stock biomass remains relatively high. This corresponds to the OSY outcome at a zero discount rate. Consequently, MEY represents a more appropriate management target for the fishery, even though it may imply lower catch levels compared to MSY. The resulting gains in stock biomass and economic efficiency justify its adoption as a preferred reference point.

The findings further indicate that observed fishing effort levels exceed the MEY effort. Management interventions should therefore focus on reducing fishing effort to sustainable levels. Strengthening enforcement of fisheries regulations through regular monitoring and patrols can contribute to effort reduction. In addition, involving local leaders in co-management arrangements may improve compliance and effectiveness of management measures. Efforts should also be directed toward stock recovery through measures such as extending closed seasons to allow for adequate biological regeneration.

## Conclusions

The declining CPUE indicates that the fishery is currently being exploited at an unsustainable rate, making the situation ecologically unsound. This implies that fishing effort has been excessively high. Consequently, analysis of the four reference points (MSY, MEY, OAY, and OSY), together with the bifurcation analysis, shows that the optimal fishing effort is 444.7 (approximately 445 trips per year), which corresponds to the MEY reference point. This effort level ensures the highest fish stock biomass and maximum sustainable net revenue (profit). Because all observed effort values exceed 445, there is a need to implement measures to reduce fishing effort. Potential interventions include introducing property rights, applying tax policies, or extending the closed season. The OSY reference point further indicates that increasing the discount rate raises effort and harvest (yield), while reducing fish stock (biomass) and sustainable net revenue (profit). OSY levels converge to MEY at a zero discount rate and to OAY at an infinite discount rate. The bioeconomic modelling approach used in this study may be extended to other data-limited fisheries in the region, where integrated bioeconomic and bifurcation analyses can provide useful guidance for sustainable exploitation and management.
